# Short-term assessment of pain and discomfort during rapid maxillary expansion with tooth-bone-borne and tooth-borne appliances: randomized clinical trial

**DOI:** 10.1590/2177-6709.28.4.e2322220.oar

**Published:** 2023-09-15

**Authors:** Bruno de Paula Machado PASQUA, Cristiane Barros ANDRÉ, João Batista de PAIVA, José RINO

**Affiliations:** 1 University of São Paulo, School of Orthodontics (São Paulo/SP, Brazil).; 2 University of Mogi das Cruzes, Technology Research Center (Mogi das Cruzes/SP, Brazil).; 3 University of São Paulo, Department for Orthodontics (São Paulo/SP, Brazil).

**Keywords:** Rapid palatal expansion, Orthodontic anchorage procedures, Pain

## Abstract

**Objective:**

The aim of this randomized clinical trial was to evaluate and compare, during the first week of rapid maxillary expansion (RME), the impact caused by two types of appliances: Hyrax and Hybrid Hyrax.

**Methods:**

Forty-two patients who met the eligibility criteria (aged 11-14 years, with transverse maxillary deficiency, posterior crossbite, and presence of maxillary first premolars and first permanent molars) were selected and randomly divided into two groups: TBB GROUP (tooth-bone-borne expander), treated with Hybrid Hyrax (12 females and 9 males, mean age 13.3 ± 1.3 years), and TB GROUP (tooth-borne expander), treated with Hyrax (5 females and 16 males, mean age 13.3 ± 1.4 years). Pain and discomfort were assessed in two times: after the first day of activation (T1) and four days after, by means of the numerical rate scale and the instrument MFIQ (Mandibular Functional Impairment Questionnaire). Descriptive statistics and the Mann-Whitney test were used for comparison between groups and between sexes. A 5% significance level was adopted.

**Results:**

Both appliances had a negative impact, generating pain and discomfort, and reducing functional capacity. However, the scores obtained were of low intensity and no significant differences were observed between the groups. Considering sexes, there were statistically significant differences, with the female sex presenting higher scores for pain and functional limitation.

**Conclusions:**

Despite causing impact in pain and increase in the functional limitation, these changes were of low intensity, with no statistical difference between the groups. Females were more sensitive to the impact caused by the RME.

## RESUMO

**Objetivo:**

O objetivo deste ensaio clínico randomizado foi avaliar e comparar, durante a primeira semana de expansão rápida da maxila (ERM), o impacto causado por dois tipos de aparelhos: Hyrax e Hyrax Híbrido.

**Métodos:**

Quarenta e dois pacientes que atendiam aos critérios de seleção (idade de 11 a 14 anos, com deficiência transversal da maxila, mordida cruzada posterior e presença de primeiros pré-molares e primeiros molares permanentes superiores) foram selecionados e divididos aleatoriamente em dois grupos: Grupo DOS (expansor dento-osseossuportado), tratado com Hyrax Híbrido (12 mulheres e 9 homens, idade média 13,3 ± 1,3 anos), e Grupo DS (expansor dentossuportado), tratado com Hyrax (5 mulheres e 16 homens, idade média de 13,3 ± 1,4 anos). A dor e o desconforto foram avaliados em dois momentos: após o primeiro dia de ativação (T1) e após quatro dias, por meio da escala de frequência numérica e do instrumento MFIQ (Questionário de Limitação Funcional Mandibular). A estatística descritiva e o teste de Mann-Whitney foram utilizados para comparação entre os grupos e entre os sexos. Adotou-se nível de significância de 5%.

**Resultados:**

Ambos os aparelhos tiveram impacto negativo, gerando dor e desconforto e reduzindo a capacidade funcional. No entanto, os escores obtidos foram de baixa intensidade e não foram observadas diferenças significativas entre os grupos. Considerando os sexos, houve diferenças estatisticamente significativas, com o sexo feminino apresentando maiores escores para dor e limitação funcional.

**Conclusões:**

Apesar de causar impacto na dor e aumento na limitação funcional, essas alterações foram de baixa intensidade, sem diferença estatística entre os grupos. As mulheres foram mais sensíveis ao impacto causado pela ERM.

## INTRODUCTION

Rapid maxillary expansion (RME) is a procedure that aims to correct maxillary transverse deficiency and posterior crossbite by opening the midpalatal suture. This technique has proven effective in orthodontics, and is commonly used in clinical practice.^1^ ^,^ ^2^ Although, some side effects, such as buccal tipping of posterior teeth, root resorption of supporting teeth, and changes in buccal and palatal bone plate thickness of maxillary premolars, have been observed with both tooth-tissue-borne and tooth-borne appliances.^3^ ^-^ ^5^

Wilmes et al.^6^ developed a tooth-bone-borne expander for growing patients, with the goal of potentiating orthopedic effects and decreasing side effects during RME. This appliance has hybrid support: posterior dental support and anterior support provided by means of orthodontic mini-screws in the palatal region, located posteriorly to the third palatal rugae. This appliance is advantageous in performing RME for patients with unerupted premolars and absent or incomplete root development; additionally, it provides more pronounced skeletal changes, minor side effects in the first premolar region, less tooth tipping, and low impact on the oral health-related quality of life.^7^ ^-^ ^9^ Besides that, an important finding was the more pronounced effect in the nasal region,^9^ ^,^ ^10^ suggesting a greater increase in airway volume compared to conventional appliances.^11^

Patients undergoing RME with conventional appliances often report discomfort, pain, and even functional limitations.^12^ ^-^ ^14^ However, few studies have specifically evaluated the effects of the Hybrid Hyrax. A recent study reported no significant differences in pain and discomfort between Hyrax and Hybrid Hyrax.^15^

Several methods for measuring pain intensity have been described in the literature, and the pain numerical rate scale (NRS) has proven to be more appropriate due to the ease of clinical application and patient understanding.^16^ For the evaluation of discomfort, quality of life, as well as functional limitations, there are psychometric instruments specific for Dentistry.^17^ ^-^ ^19^ The mandibular functional impairment questionnaire (MFIQ) specifically aims to assess the patient’s perception of mandibular functional impairment, such as difficulty in eating, speaking, swallowing, and yawning.^18^ ^,^ ^19^

### SPECIFIC OBJECTIVES OR HYPOTHESES

Considering the importance of patients’ well-being, the present study aimed to evaluate and compare the impact of two types of maxillary expansion appliances (tooth-bone-borne and tooth-borne) with respect to pain, discomfort, and functional limitation during the first week of RME activation in growing patients, by assessing pain (NRS) and functional limitation (MFIQ). Since the Hybrid Hyrax is a new appliance, the literature on its symptomatology is scarce. Although this appliance has shown promising results, it involves a more invasive technique than traditional appliances, then it is necessary to understand more broadly its impact. The null hypothesis tested was that there would be no difference for the pain and discomfort impact between these appliances.

## MATERIAL AND METHODS

### ETHICAL ASPECTS AND STUDY DESIGN

This was a prospective randomized clinical trial that was approved by the Ethics Committee on Human Research of University of São Paulo, School of Dentistry, under the protocol number: 3.311.813. This study was also registered in the REBEC clinical trials (RBR-48g9q6). The Consolidated Standards of Reporting Trials (CONSORT) statement and guidelines were followed.

### PARTICIPANTS, ELIGIBILITY CRITERIA, AND SETTING

Patients aged 11-14 years, who visited the orthodontic clinic at University of São Paulo, School of Dentistry between January and July 2018, were screened for eligibility. Participants who met the eligibility criteria were invited to participate, and informed consent was obtained from all patients and their parents or legal guardians. The inclusion criteria were as follows: age between 11 and 14 years, transverse maxillary deficiency, bilateral or unilateral posterior crossbite, and the presence of maxillary first premolars and maxillary first permanent molars. The exclusion criteria were: the presence of systemic diseases, history of previous orthodontic treatment, presence of cleft lip and palate, presence of congenital deformities, and agenesia or loss of permanent teeth. 

### INTERVENTIONS

The Hybrid Hyrax appliance used in this study was supported by two mini-implants inserted in the anterior region of the palate, posterior to the third palatal rugae, paramedian 2-3 mm from the palatal raphe, based on the appliance of Wilmes et al.^6^ This site, known as the T-zone, has great bone thickness and density, and is located away from structures such as roots, blood vessels, or nerves.^20^ ^,^ ^21^ The mini-implants were placed manually. To obtain the correct angulation, a mini-implant hand-key was used (Peclab, Belo Horizonte/MG), with fitting for counter-angle (Kavo do Brasil Ind. Com. Ltda, Joinville/SC, Brazil). The upper first permanent molars were chosen as posterior anchorage and banded. 

**Figure 1:**
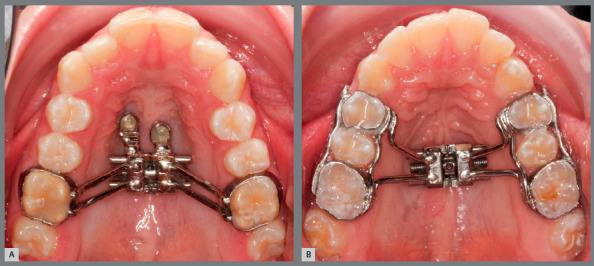
A) TBB group ( Hybrid Hyrax ). B) TB group ( Hyrax ).

Mini-implants (1.5-mm in diameter; 8-mm in length, Dental Morelli LTDA, Sorocaba/SP, Brazil) were inserted after local anesthesia using lidocaine. Further, a digital dental scan of the maxillary arch was performed using an intraoral scanner (Trios Pod version, 3Shape, Copenhagen, Denmark). The model was printed using a Form2 printer (Form labs, Somerville, Massachusetts, USA), and the appliance was fabricated on the printed model (Fig 1A, Hybrid Hyrax, tooth-bone-borne appliance, TBB group).

The same digital workflow was used to manufacture the Hyrax tooth-borne appliance (TB group, Fig 1B), which was anchored on four bands (first premolars and first molars). For both groups, the 11-mm Hyrax-type expander screw (Peclab, Belo Horizonte, Minas Gerais, Brazil) was used.

All patients were treated by the same orthodontist, and the activation protocol was the same in both groups: The expander screw was activated on the first day with one full turn (four activations of ¼ turn), and in the following days, ¼ turn twice a day (every 12 h) until correction of the maxillary deficiency and overcorrection of crossbite (occlusion of the palatal cusp of the maxillary first permanent molars with the corresponding buccal cusp of the mandibular first permanent molars). No analgesics were prescribed; however, the patients were allowed to use them at their discretion. None of the patients reported using analgesics.

### MEASUREMENTS

####  Pain intensity assessment 

**Figure 2:**
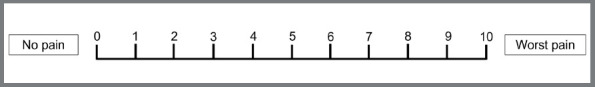
Numerical rate scale ( NRS ) for pain assessment.

The pain NRS (Fig 2) was used for the subjective assessment of pain intensity experienced by the patients (Table 1). The participants scored the pain in different regions of the mouth (Table 1) using a numerical scale from 0 to 10, based on the article by Feldman and Bazargani.^15^ ‘No pain’ was scored as 0, and ‘The worst possible pain’ was scored as 10. 

**Table 1:** Questions concerning pain and discomfort, assessed at T1 (after the first day of activation) and T2 (after the fourth day of activation) (Feldman and Bazargani^15^, 2017).

Parameter	Score (0 to 10)
PAIN	
1- Do you now have pain?	
2 - Do you now have pain from the molars?	
3 - Do you now have pain from the incisors?	
4 - Do you now have pain from the upper jaw?	
5 - Do you now have pain from the palate?	
6 - Do you now have pain from the tongue?	
DISCOMFORT	
7 - Do you experience tensions in your upper jaw?	
8 - Do you experience tensions in your teeth?	
9 - Do you experience soreness from the appliance?	

####  MFIQ instrument 

Using the MFIQ,^19^ it was possible to quantify the patient’s functional limitations regarding functional capacity and eating. The original version comprised 17 items. In the present study, the Portuguese validated version was used.^18^ The instrument was applied using an interview in the first week of activation at two time-points (T1 - after the first day of activation, and T2 - after the fourth day of activation), according to the methodology of Feldmann and Bazargani.^15^ A score was assigned to each question that represented the level of difficulty to develop routine activities, ranging from 0 (no difficulty) to 4 (very difficult or impossible without help). The creators of this instrument have proposed the possibility of categorizing the results in quantitative (ranging from 0 to 1) and qualitative (low, moderate, or severe functional impairment) formats. A quantitative format was used to facilitate the data interpretation. 

### PRIMARY OUTCOME

The primary outcome was the comparison between groups and sexes regarding pain intensity, discomfort, and functional limitation during the first week of RME activation with the two appliances evaluated.

The secondary outcome was the correlation between pain and MFIQ with age and skeletal maturation of the midpalatal suture.

### SAMPLE SIZE CALCULATION

This study used the same sample as well as some statistical data of a previous randomized clinical trial.^9^ However, other parameters were evaluated using new information. The present study aimed at evaluating dental and skeletal effects of RME, using cone-beam computed tomography (CBCT). A sample calculation was performed based on skeletal changes after RME, observed on the coronal section of CBCT images, specifically in the premolar region,^7^ reported as being on average equal to 3.33 ± 3.58 mm. Considering a significance level of 0.05 and a type II error of 20%, the minimum number of patients per group was calculated to be 19, using a two-tailed test. Considering a sample loss of 10%, the final sample size was calculated as 42, with 21 patients per group.

### INTERIM ANALYSES AND STOPPING GUIDELINES

No interim analysis was conducted, all data were analyzed after the study was completed.

### RANDOMIZATION

The sequence of 42 numbers corresponding to the patients (each number corresponding to a patient) was randomized into two groups using the excel RANDOM function.

### BLINDING

Double blinding was not possible due to the type of interventions administered (clinical treatment). However, before the data assessment and statistical analysis, the questionnaires were identified with only a coded ID number, for another examiner to compute the scores. Therefore, the examiner did not know which patient the scores belonged to.

### STATISTICAL ANALYSIS

The evaluated measurements were described according to groups, using means ± standard deviations, or medians and interquartile ranges, and the values before expansion were compared between the groups using Student’s t-test or Mann-Whitney U test. The sex of the patients was described according to groups, using absolute and relative frequencies; and the association between the groups was determined using chi-square or Fisher’s exact tests.^22^

For the comparison of pain and functional limitation between the groups and between sexes, the Mann-Whitney U test was used.^22^ Thus, for pain and the total value, each parameter was evaluated separately (region of pain and discomfort). To interpret the MFIQ instrument, the raw score of each of the two domains was analyzed individually; the patient’s total functional limitation was analyzed following the methodology of Stengenga et al.^19^ (calculation of the raw score component, which ranges from 0 to 1). This comparison was performed between T1 and T2. For all the intergroup comparisons, the observed power was calculated by the Student’s t-test, to present the sample’s power of discrimination on the results.^22^

Spearman’s correlations were calculated between pain and MFIQ and the data regarding the initial age and midpalatal suture maturation (evaluated by the method of Angelieri et al.^23^) to verify possible correlations between them. Differences with a *p*-value of less than 5% (*p*< 0.05) were considered statistically significant. Analyses were performed using IBM SPSS for Windows v. 20.0 (SPSS Inc., Chicago, IL, USA).

## RESULTS

### PARTICIPANTS FLOW

**Figure 3:**
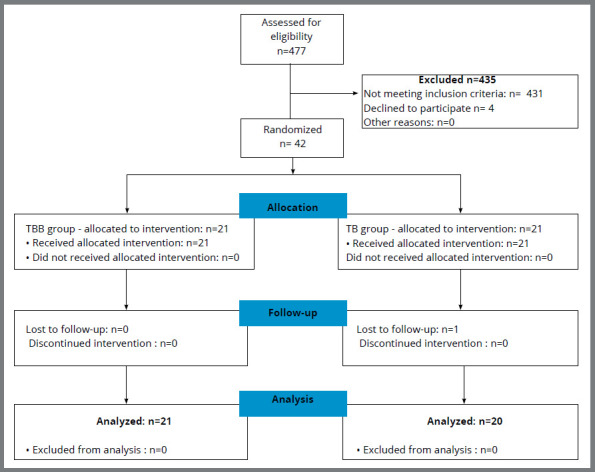
Consort flow chart diagram.

A total of 477 patients were screened between January and July 2018. By means of clinical examination, 42 participants were enrolled; 431 participants were excluded because they did not meet the eligibility criteria, and four dropped out (Fig 3). After the recruitment, forty-two patients were randomly assigned to the study groups in a 1:1 ratio. Only one patient was lost because he/she missed the appointment and did not answer the questionnaires (Fig 3). TBB group was composed by 12 girls and 9 boys, with mean initial age of 13.3 years, and TB group was composed by 5 girls and 16 boys with mean initial age of 13.2 years.

### BASELINE DATA

Table 2 shows that the sexes distribution between groups was statistically different (*p*= 0.037). Regarding the other initial characteristics, there were no statistically significant differences between the groups.

**Table 2:** Baseline characteristics of the groups.

Parameter	TBB group Hybrid Hyrax Mean (SD)	TB group Hyrax Mean (SD)	TBB - TB Mean difference (95% CI)	P value
Sex				0.037^†^*
Female (%)	12 (57.1%)	5 (23.8%)		
Male (%)	9 (42.1%)	16 (76.2%)		
Midpalatal suture maturation				0.911^§^
Stage A	1 (2.4%)	3 (7.1%)		
Stage B	6 (14.3%)	3 (7.1%)		
Stage C	7 (16.7 %)	7 (16.7 %)		
Stage D	3 (7.1%)	4 (9.5%)		
Stage E	4 (9.5%)	4 (9.5%)		
Age (years)	13.3 (1.3)	13.3 (1.4)	0 (-0.7; 0.9)	0.782 ^‡^

SD: standard deviation. CI: confidence interval. ^†^ *p-*values for Pearson’s chi-square test. ^§^ *p*-values for Mann-Whitney test. ^‡^ *p-*values for independent *t*-test. *p<0.05; **p<0.01; ***p<0.001; ****p<0.0001.

### NUMBERS ANALYZED FOR EACH OUTCOME, ESTIMATION, AND PRECISION

No statistically significant differences were found between the groups for any of the questions evaluated, regarding pain and discomfort, at T1 and T2 (Table 3).

**Table 3:** Medians, percentile range, *p-*value and observed power resulting from comparative analysis for pain. Comparisons between groups defined by Mann-Whitney, and significance at *p*<0.05.

	TBB Md (IQR)	TB Md (IQR)	*p*	Observed Power	TBB Md (IQR)	TB Md (IQR)	*p*	Observed Power
	T1				T2			
PAIN								
1 - Do you now have pain?	2 (0; 3)	0.5 (0; 2.3)	P= 0.4263	0.096	0 (0; 2)	0 (0; 1)	P= 0.225	0.297
2 - Do you now have pain from the molars?	2 (0; 4)	0.5 (0; 3)	P= 0.4039	0.126	1 (0; 3)	0 (0; 1.3)	P= 0.187	0.227
3 - Do you now have pain from the incisors?	0 (0; 2)	0 (0; 0)	P= 0.2849	0.208	2 (0; 5)	0 (0; 1.3)	P= 0.078	0.633
4 - Do you now have pain from the upper jaw?	1 (0; 2)	1 (0; 1.3)	P= 0.7842	0.091	0 (0; 3)	0 (0; 2.3)	P= 0. 403	0.111
5 - Do you now have pain from the palate?	0 (0; 0)	0 (0; 1)	P= 0.5661	0.056	0 (0; 1)	0 (0; 0)	P= 0.210	0.270
6 - Do you now have pain from the tongue?	0 (0; 0)	0 (0; 1)	P= 0.5144	0.152	0 (0; 0)	0 (0; 2)	P= 0.575	0.062
DISCOMFORT								
7 - Do you experience tensions in your upper jaw?	3 (1; 5)	2 (0; 4.3)	P= 0.2405	0.220	2 (0; 6)	1.5 (0.8; 3.3)	P= 0.522	0.225
8 - Do you experience tensions in your teeth?	4 (2; 6)	3 (1; 4.3)	P= 0.3613	0.186	5 (1; 9)	2.5 (1; 5)	P= 0. 170	0.446
9 - Do you experience soreness from the appliance?	4 (0; 5)	2 (0.8; 3.5)	P= 0.4339	0.167	4 (1; 8)	2 (0; 3)	P= 0.215	0.461
TOTAL SCORE	2.2 (1.5; 2.8)	1.7 (1.1; 2.3)	P= 0.205	0.169	2.5(1.6; 3.4)	1.4 (0.7; 2.1)	P= 0.066	0.502

Md: median. IQR: interquartile range. *p<0.05; **p<0.01; ***p<0.001; ****p<0.0001.

**Figure 4:**
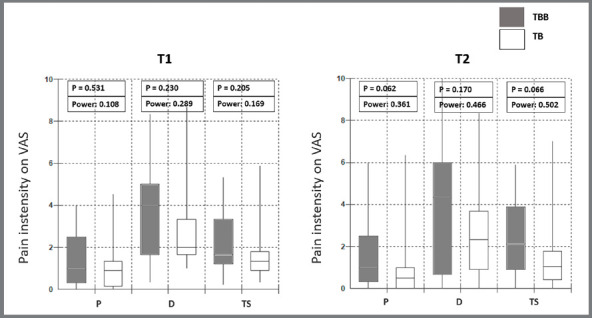
Median values, percentile ranges, and observed power, concerning to pain intensity (P), discomfort (D) and total score (TS) related to RME in the first week in treatment.

Regarding the analysis of pain and discomfort, there was no statistically significant difference between the groups (Fig 4).

According to the intergroup comparison of MFIQ results, there was no statistically significant difference between the groups in terms of functional capacity, feeding, and functional limitation (Table 4). For the comparative analysis between sexes (Table 5), no statistically significant differences were found between male and female with respect to total pain. However, for functional capacity, nutrition, and total MFIQ, greater sensitivity was found in females, with statistically significant differences.

**Table 4:** Medians, interquartile range, *p-*value and observed power resulting from comparative analysis for Functional capacity, Feeding, and MFIQ total score. Comparisons between groups defined by Mann-Whitney and significance at p<0.05.

	TBB Md (IQR)	TB Md (IQR)	P	Observed Power	TBB Md (IQR)	TB Md (IQR)	P	Observed Power
	T1				T2			
Functional capacity (FC)	0.3 (0.1; 0.7)	0.3 (0; 0.7)	0.9480	0.053	0.3 (0; 0.7)	0.2 (0; 0.6)	0.592	0.130
Feeding (F)	0.6 (0; 1)	0.4 (0; 1)	0.6481	0.012	0.5 (0; 1)	0.4 (0; 1)	0.396	0.130
MFIQ total score	0.5 (0; 0.8)	0.4 (0.2; 0.8)	0.8756	0.059	0.43 (0; 0.8)	0.3 (0; 0.7)	0.433	0.149

Md: median. IQR: interquartile range. *p<0.05; **p<0.01; ***p<0.001; ****p<0.0001.

**Table 5:** Medians, interquartile range, *p-*value and observed power resulting from comparative analysis for Functional capacity, Feeding, and MFIQ total score. Comparisons between genders defined by Mann-Whitney and significance at p<0.05.

	Male Md (IQR)	Female Md (IQR)	P	Observed Power	Male Md (IQR)	Female Md (IQR)	P	Observed Power
	T1				T2			
Pain	1.2 (0.7; 2.2)	1.7 (1.1; 3.2	0.098	0.375	0.6 (0.2; 2.4)	1.7 (0.9; 3)	0.100*	0.354
Functional capacity	0.2 (0.1; 0.3)	0.4 (0.3; 0.5)	0.031*	0.495	0.1 (0.1 0.4)	0.3 (0.2; 0.4)	0.025*	0.492
Feeding	0.5 (0.3; 0.6)	0.7 (0.5; 0.8)	0.010*	0.762	0.3 (0.1; 0.7)	0.5 (0.4; 0.7)	0.027*	0.600
MFIQ total score	0.3 (0.2; 0.4)	0.5 (0.4; 0.6)	0.005**	0.761	0.2 (0.1; 0.5)	0.4 (0.3; 0.5)	0.011*	0.643

Md: median. IQR: interquartile range. *p<0.05; **p<0.01; ***p<0.001; ****p<0.0001.

Finally, no significant correlations were found between pain and MFIQ and age and maturity of the midpalatal suture at both T1 and T2 (Table 6).

## DISCUSSION

Orthodontic patients frequently report pain and discomfort.^24^ Few studies have reported these manifestations in RME.^1^ ^-^ ^14^ ^,^ ^24^ The RME expanders are well-accepted by patients, despite the common reports of pain. Studies of these side effects in patients treated with tooth-bone-borne expansion appliances are less frequent.^15^ In addition to analyzing the efficacy of a new treatment method, it is also necessary to investigate the patients’ acceptance and adaptation to the new appliance, especially the impact of pain, eating discomfort, and the patient’s functional capacity. Efficient care is necessary for managing these signs and symptoms, which are common during RME. 

Common methods to assess patients’ experiences of pain during treatment include the use of pain scales. The visual analog scale and the NRS are the most commonly used.^25^ In the present study, the numerical scale was chosen, since it has already been presented as a method of easy applicability and understanding by the patient.^16^ To evaluate the experience of pain specifically for RME, the methodology described by Feldemann and Bazargani was used,^15^ since it was the only study that aimed to score the pain directed to the areas most commonly affected by RME. 

The assessment of pain score and the use of MFIQ instrument were performed after the first and fourth days of the first activation, since this is the time of greatest patient discomfort during orthodontic treatment (first week).^15^ ^,^ ^26^. In the present study the patients had mean age of 13.27 ± 1.32 years. The choice of this age range (11 to 14 years old) was based on other studies with Hybrid Hyrax,^6^ ^,^ ^7^ because during this period, RME indications are more sensible. Although this is still a growth phase, the midpalatal suture may be more interdigitated, becoming resistant to RME,^24^ ^,^ ^27^ and hybrid anchorage is indicated in these cases. A statistically significant difference between the groups was observed according to sex. Despite the randomness in the selection, because it is a small sample for a categorized variable, this unbalance can occur. Nonetheless, it was assumed that it did not influence the results.

Both appliances caused pain (Table 3) during the first week of activation, as well as changes in functional capacity and feeding (Table 4). However, these changes were of a low intensity. Regarding pain at T1, on a scale of 0 to 10 (considering the total score), the medians (percentiles) were 1.7 (1.2-3.3) in the TBB group, and 1.3 (0.9-1.8) in the TB group, with no statistically significant difference between the groups. At T2, the medians (percentiles) were 2.1 (0.9-3.9) in the TBB group and 1.1 (0.4-1.8) in the TB group, with no statistically significant difference. Considering the different regions assessed, the most common pain was general pain (question 1 - Table 3), and pain in the molar region (question 2 - Table 3). This occurred in both groups and may be a consequence of the appliance support, which in both groups occured in the first permanent molars. No statistically significant difference was observed between the groups in any of the variables (questions) evaluated. This indicates that both appliances are well-tolerated by patients, with respect to pain. This is an important finding when considering RME treatment anchored on miniscrews, since the advantages of these appliances, such as better skeletal outcomes, better outcomes in terms of increased skeletal changes, and fewer dental side effects, have already been observed.^9^ Nevertheless, a more pronounced sensitivity was found in those patients treated with the Hybrid Hyrax, unlike what was previously reported.^15^ This also occurred regarding discomfort, in question 9 (Table 3), in which the Hybrid appliance showed twice the value of the Hyrax. Despite this discrepancy, this raises an alert that patients treated with Hybrid Hyrax may have a slight increase in sensitivity during rapid maxillary expansion.

Additionally, in both groups, the intensity of pain was lower at T2. Pain during RME is reportedly greater in the first activation, whereas in the study of Halicioğlu et al.,^13^ the peak of pain was at the fifth activation, and in the study of Nedlemann et al.,^12^ it was at the sixth activation. In the present study, a higher peak of pain was found at T1, which coincides with the fifth and sixth activations of the appliance, which conforms with the results of these studies. This provides further evidence of the similarity between the two types of appliances in terms of pain symptoms.

Orthodontists know that with aging, bone maturation of the midpalatal suture increases.^23^ ^,^ ^27^ Thus, the authors of the present study believe that older patients experience more pain due to the greater resistance to expansion caused by the midpalatal suture, which is more interdigitated. Conversely, the results of this study showed that, considering both groups, there was no correlation between pain and age at both T1 and T2 (Table 6). This result can be explained by the short age range of patients in this study (11-14 years). The findings of the present study are consistent with those of previous studies.^12^ ^,^ ^15^ ^,^ ^28^

**Table 6:** Spearman correlation coefficient (significance at *p*<0.05).

	Initial age r(p)	Midpalatal suture maturation r(p)	Initial age r(p)	Midpalatal suture maturation r(p)
	T1		T2	
Pain	-0.029 (0.859)	0.100 (0.532)	-0.041 (0.798)	0.166 (0.301)
Functional capacity	-0.098 (0.542)	0.082 (0.611)	-0.077 (0.632)	0.061 (0.707)
Feeding	0.085 (0.597)	0.009 (0.955)	0.073 (0.651)	0.132 (0.410)
TOTAL MFIQ	-0.040 (0.804)	0.080 (0.617)	-0.016 (0.919)	0.131 (0.413)

*p<0.05; **p<0.01; ***p<0.001; ****p<0.0001.

The results showed that there were statistically significant differences between sexes, considering the variables assessed by the MFIQ instrument (Table 5), with the worst experience reported among females, which is in agreement with a previous study.^14^ Thus, this difference regarding pain between sexes should be considered during pain management in RME treatments. However, other studies have reported no statistically significant differences between groups.^12^ ^-^ ^14^

Regarding the MFIQ instrument, no statistically significant differences were found between the groups at T1 and T2 in terms of the functional capacity, nutrition, and functional limitation. The medians obtained from the total score for functional limitation in both groups were of low intensity. These results, reveal that the limitation caused by both appliances was similar, as previously reported.^15^

A greater impact was noticed in both groups at T1 than at T2. This probably occurred because the participants begin to get accustomed to the appliance and to the changes that occurred in their mouth. Despite this, the scores at T2 were lower in both groups, with no statistically significant difference between them, suggesting that the patients were adapted. Moreover, as the pain decreased concomitantly, the patients’ activities became unaltered. 

The equivalence between the symptomatology during RME and between the two evaluated appliances is extremely important data for the literature, because both appliances were well-tolerated by the patients. One should consider that the hybrid Hyrax generates a slightly higher cost, due to requiring intraoral scanning. However, considering the advantages observed in the reduction of side effects,^7^ ^,^ ^9^ ^,^ ^10^ more pronounced skeletal effects and better efficiency in nasal airway improving,^11^ the use of this appliance seems promising. Systematic reviews are essential to substantiate the findings of these studies.

## LIMITATIONS AND GENERALIZABILITY

The sample size calculation for this study was based on skeletal changes in the nasomaxillary region, and not on pain intensity or discomfort. The mini-implants insertion process can generate discomfort in the first hours after insertion, and future studies are necessary to evaluate and consider pain during mini-implants placement.

## HARMS

No serious harm was observed other than pain and discomfort during RME.

## CONCLUSIONS

- » Pain and functional limitation were common for patients in both groups during RME at both T1 (1 day after the start of activation) and T2 (4 days after the first activation). The values obtained were of low intensity, with no statistical difference between the groups.
- » There was no correlation between pain and functional limitation with age or skeletal maturation of the midpalatal suture.
- » Female patients experienced higher pain perception and functional limitations during RME.
